# Characteristics of tumour stroma in regional lymph node metastases in colorectal cancer patients: a theoretical framework for future diagnostic imaging with FAPI PET/CT

**DOI:** 10.1007/s12094-022-02832-9

**Published:** 2022-04-28

**Authors:** Meaghan Polack, Sophie C. Hagenaars, Alice Couwenberg, Walter Kool, Rob A. E. M. Tollenaar, Wouter V. Vogel, Petur Snaebjornsson, Wilma E. Mesker

**Affiliations:** 1 Department of Surgery, Leiden University Medical Center, Albinusdreef 2, 2333 ZA Leiden, Zuid-Holland The Netherlands; 2 Department of Radiation Oncology, Antoni van Leeuwenhoek Hospital, Amsterdam, Noord-Holland The Netherlands; 3 Department of Nuclear Medicine, Noordwest Ziekenhuisgroep Alkmaar, Alkmaar, Noord-Holland The Netherlands; 4 Department of Nuclear Medicine, Antoni van Leeuwenhoek Hospital, Amsterdam, Noord-Holland The Netherlands; 5 Department of Pathology, Antoni van Leeuwenhoek Hospital, Amsterdam, Noord-Holland The Netherlands

**Keywords:** Colorectal cancer, Cancer-associated fibroblasts, Tumour-stroma ratio, Lymph nodes, FAPI

## Abstract

**Purpose:**

The recently developed fibroblast activation protein inhibitor (FAPI) tracer for PET/CT, binding tumour-stromal cancer-associated fibroblasts, is a promising tool for detection of positive lymph nodes. This study provides an overview of features, including sizes and tumour-stromal content, of lymph nodes and their respective lymph node metastases (LNM) in colorectal cancer (CRC), since literature lacks on whether LNMs contain sufficient stroma to potentially allow FAPI-based tumour detection.

**Methods:**

Haematoxylin and eosin-stained tissue slides from 73 stage III colon cancer patients were included. Diameters and areas of all lymph nodes and their LNMs were assessed, the amount of stroma by measuring the stromal compartment area, the conventional and total tumour-stroma ratios (TSR-c and TSR-t, respectively), as well as correlations between these parameters. Also, subgroup analysis using a minimal diameter cut off of 5.0 mm was performed.

**Results:**

In total, 126 lymph nodes were analysed. Although positive correlations were observed between node and LNM for diameter and area (*r* = 0.852, *p* < 0.001 and *r* = 0.960, *p* < 0.001, respectively), and also between the LNM stromal compartment area and nodal diameter (*r* = 0.612, *p* < 0.001), nodal area (*r* = 0.747, *p* < 0.001) and LNM area (*r* = 0.746, *p* < 0.001), novel insight was that nearly all (98%) LNMs contained stroma, with median TSR-c scores of 35% (IQR 20–60%) and TSR-t of 20% (IQR 10–30%). Moreover, a total of 32 (25%) positive lymph nodes had a diameter of < 5.0 mm.

**Conclusion:**

In LNMs, stroma is abundantly present, independent of size, suggesting a role for FAPI PET/CT in improved lymph node detection in CRC.

## Introduction

Colorectal cancer (CRC) is the third most common cancer type worldwide, with high morbidity and mortality rates [1–3]. In the diagnostic and preoperative workup, imaging with CT and/or MRI is performed for local, i.e. the primary tumour and regional lymph nodes, and distant extent of the disease. For clinical lymph node staging, positive lymph nodes are identified using size cut-off values and other morphological characteristics, such as roundness or irregular borders, according to radiological guidelines using these standard anatomical imaging techniques [4–8]. The detection of actual lymph node metastases (LNMs) remains suboptimal, however, since false negative outcomes are prevalent, affecting treatment selection [9]. Therefore, other imaging modalities, e.g. FDG PET/CT or functional MRI, are being studied, but have not yet led to improved accuracy for detecting LNMs in CRC patients [10, 11].

Based on recent developments in the field of the tumour microenvironment, a novel PET/CT tracer has been developed: the fibroblast activation protein inhibitor (FAPI), which can be conjugated to a radioactive isotope such as Gallium-68 or Fluorine-18. The FAPI tracer binds to a part of the tumour microenvironment, or tumour stroma, which plays an essential role in tumour behaviour and the metastasis process. This tumour stroma is mainly composed of lymphocytes, vasculature, the extracellular matrix and fibroblasts. A desmoplastic reaction of constant remodelling by collagenases and matrix metalloproteases is associated with an epithelial-to-mesenchymal transition [12, 13]. This process not only promotes tumorigenesis, but also activates the quiescent fibroblasts in various ways, giving rise to the so-called cancer-associated fibroblasts (CAFs). These CAFs express the fibroblast activation protein (FAP), to which the FAPI can bind specifically [13–15].

So far, literature has shown promising results with FAPI PET/CT in cancer patients, suggesting improved sensitivity and specificity compared to the FDG PET/CT [16–21]. In addition to good uptake in the primary tumour, a high tumour-to-background ratio is seen with the FAPI tracer, leading to better imaging of regional and distant extent of disease. Furthermore, no specific patient protocols are necessary to prevent errors in imaging, e.g. fasting or regulation in medicine, due to the characteristics in biodistribution and the pharmacokinetics of FAPI [20, 22–26].

Over the past decades, the tumour stroma has been subject to extensive research. The tumour-stroma ratio (TSR), standardized and validated for primary tumours in multiple retrospective studies, expresses the proportional size of the stromal compartment relative to the epithelial tumour cell compartment and has proven to be of significant prognostic and predictive value in various types of carcinomas [13, 27–30]. Stromal abundance in primary tumours is herein associated with a worse overall survival (OS) and disease-free survival (DFS), including in colon cancer [31–35]. In addition to the metastasized tumour epithelial cells, a LNM often also contains tumour stroma. Patients with a high amount of stroma in their LNMs, especially when combined with a stroma-high primary tumour, were observed to have an even worse OS and DFS than patients with stroma-low tumours and/or LNMs. Studies have shown that the TSR in biopsies correlate to the TSR score in resection material [36]. However, no information between the TSR scores between the primary tumour and their LNMs exists, necessitating examination of all positive lymph nodes for accurate diagnosis and prognosis [37–39].

In theory, the FAPI tracer binds to CAFs in the primary tumour as well as to CAFs in the LNMs, which is potentially visualized by a PET/CT scan. Although the FAPI PET/CT scanning is being studied more and more in depth and imaging recently has distinguished different pathological cancer types based on uptake, however, it is still unknown what the quantification is of tumour stroma in the LNMs in CRC patients, and thus whether small positive lymph nodes and their LNM contain enough stroma for uptake to potentially be detected with imaging [40]. The aim of this work is therefore to determine the histopathological features of the positive lymph nodes, their respective LNMs and especially the tumour stroma within, in stage III CRC patients, to be used as a theoretical framework. Furthermore, the correlation between the lymph node and LNM size and stromal amount will be assessed, as well as a subgroup analysis in small positive lymph nodes with a diameter of less than 5.0 mm.

## Materials and methods

### Study population

The study cohort included 73 patients diagnosed with colon carcinoma between 1996 and 2011 at the Leiden University Medical Center, the Netherlands, described in more detail in a previous study [37]. Patients underwent complete surgical resection of the primary tumour including regional lymph nodes, and all had a histologically proven stage III adenocarcinoma or mucinous adenocarcinoma. Of these 73 patients, 5 µm haematoxylin and eosin (H&E)-stained tissue sections of all the resected associated regional lymph nodes were selected. The final slides were scanned with a 3D-Histech scanner and analysed digitally using CaseViewer (version 2.4). Exclusion followed in the case of negative lymph nodes, fragmented tissue or poor quality of the digital slides. Patient samples were previously handled in a coded fashion, according to national ethical guidelines (“Code for Proper Secondary Use of Human Tissue”, Dutch Federation of Medical Scientific Societies) [37].

### Histopathological analysis

Positive lymph nodes were separately analysed by two investigators (MP, SH). Several histopathological measurements retaining to the lymph nodes and the LNM themselves were assessed, as listed and described in detail in Table 1. The amount of tumour stroma within the LNM was assessed in two ways, i.e. by determining *proportion*, expressed in percentages with the TSR, and by measuring the *absolute* size of the stromal compartment, expressed in mm^2^. The TSR was analysed using the conventional TSR scoring method (TSR-c) first, according to the requirements as described by van Pelt et al. [29], after which the positive lymph nodes were subsequently categorized as stroma-low (≤ 50%) or stroma-high (> 50%). Then, the total stromal percentage of this whole lymph node (TSR-t) was scored by eyeballing. The TSR-t was further categorized as < 5% stroma in the whole LNM, 5% stroma, and then per tenfold (i.e. 10%, 20% etc.). Consensus was reached that for the TSR-t, the cut-off value would be 5%, as this was deemed sufficient to potentially be detected by pathologists and imaging. Stromal percentages were also independently scored by the two investigators (MP, SH), maintaining a maximum of 10% difference in score for optimal interobserver agreement. If scores differed more than 10%, the case was discussed until agreement was reached. If necessary, a third observer, an experienced pathologist (PS), was decisive.

**Table 1:** Measurements of the positive lymph nodes

Variable (unit)	Description
Diameter positive lymph node (mm)	The largest diameter of the complete positive lymph node
Diameter LNM (mm)	The largest diameter of the metastatic lesion within the positive lymph node
Area positive lymph node (mm^2^)	The area of the complete positive lymph node, including the capsule and all directly adjacent lymphoid tissue and vessels within the contour of the lymph node
Area LNM (mm^2^)	The area of the largest continuous metastatic lesion in the positive lymph node, or, in case of multiple smaller lesions, including the adjacent metastatic lesions (i.e. lesions for which the distance between two lesions is less than the size of the lesion themselves)
Ratio area LNM: lymph node (%)	The ratio of the area of the metastatic lesion and the complete positive lymph node, portraying the percentage that the metastasis occupies of the lymph node
TSR-c (%)	The ratio of the stromal percentage in comparison to the tumour epithelial percentage within the metastatic lesion, scored in a 3.1 mm^2^ annotation, and meeting the requirements of van Pelt et al.
TSR-t (%)	The absolute amount of stroma in the metastatic lesion, taking in account all tissue types within the metastasis, e.g. necrosis or mucin, and categorized in percentages starting at 0–1%, 1–5% and then per tenfold (i.e. 10%, 20%, etc.)
TSR-c category (no.–perc.)	
-Stroma-low -Stroma-high -Not applicable	≤ 50% stroma > 50% stroma In the case the official 3.1 mm^2^ annotation area could not meet the requirements and an adjusted annotation size is used
Area adjusted TSR-c annotation (mm^2^)	The area of the annotation used for scoring the TSR-c in the case the official 3.1 mm^2^ annotation area could not meet the requirements
Area of stroma in LNM (mm^2^)	A feature portraying the area of the stromal compartment in the metastasis, calculated through the formula: area stroma in metastasis = (Percentage stroma/100) × Area metastasis)

*LNM* lymph node metastasis; *TSR-c* tumour-stroma ratio—conventional scoring method; *TSR-t* tumour-stroma ratio—total percentage of stroma

In evaluating the correlation between size of the positive lymph node and the corresponding LNM and stromal presence, subgroup analysis was performed using a cut-off value for lymph node diameter of 5.0 mm and a corresponding area of 19.6 mm^2^ (¼ × *π* × diameter^2^). This cut off corresponds with the smallest radiological cut-off value that is clinically applied; in accordance with the Dutch CRC guideline and previous histopathological studies, imaging criteria for LNM include a short axis diameter of ≥ 5.0 mm and/or different morphological features, like roundness [41, 42].

### Statistical analysis

Analyses are reported for the total group of analysed lymph nodes. All continuous variables were not normally distributed and therefore shown as medians with interquartile ranges (IQR). Categorical variables are presented as absolute numbers with the respective percentages. Cohen’s kappa was used for interobserver agreement in stromal scores. Correlations were evaluated between lymph node and their LNM size and stromal percentages, using the Pearson correlation coefficient. Two-tailed *p* values of less than 0.05 were considered statistically significant. All statistical analyses were conducted using the IBM SPSS Statistics version 25.0.

## Results

### Patient population and lymph nodes

**Fig. 1:**
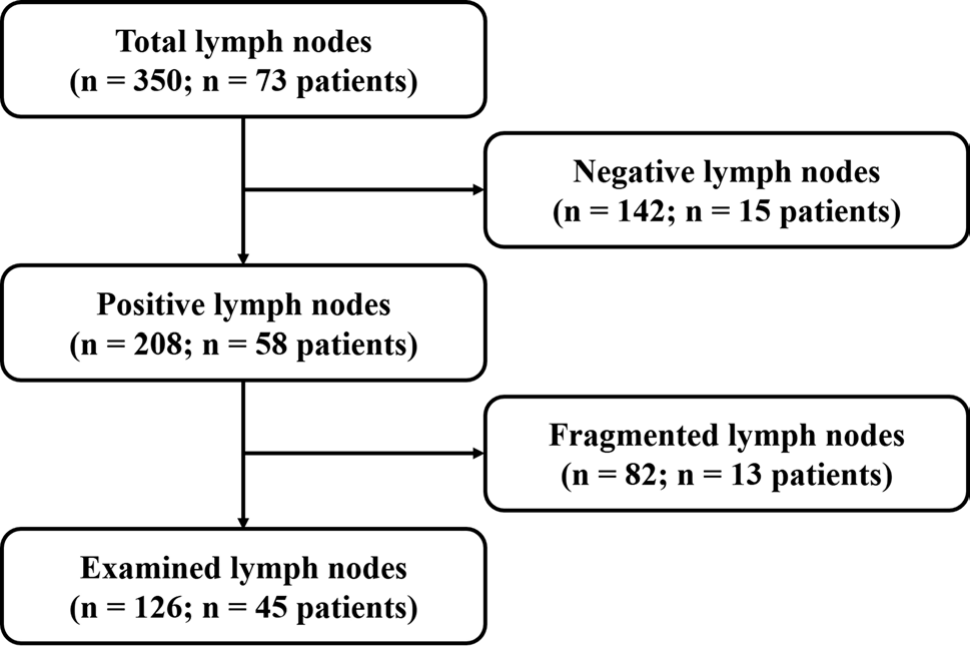
Flowchart showing the selection of analysable lymph nodes

**Fig. 2:**
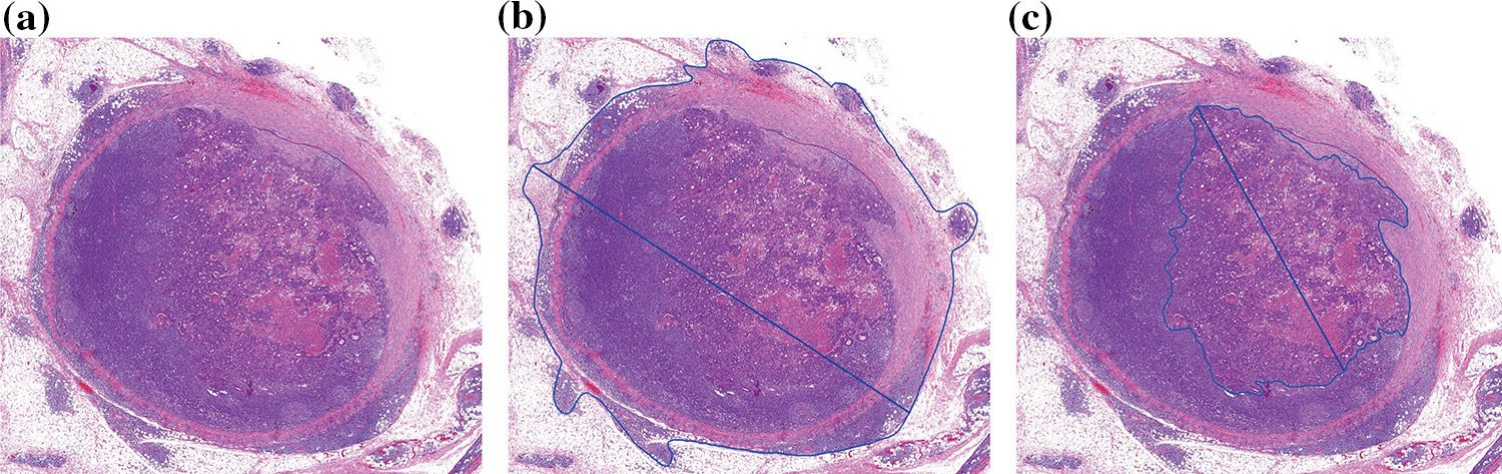
Positive lymph node containing a LNM: **a** General overview. **b** Measurement of the diameter and area of the complete lymph node. **c** Measurement of the diameter and area of the LNM

Patient characteristics are summarized in Table 2. The cohort of 73 patients had a total 350 available resected lymph nodes, of which 208 (59%) were positive and thus contained a LNM. Of these positive lymph nodes, 82 (23%) were fragmented and consequently excluded. Finally, 45 patients with 126 evaluable positive lymph nodes were included in the analysis. A flowchart of the selection of analysed nodes is shown in Fig. 1, and an overview of the measured features is provided in Table 3. In general, it was noticeable that the LNMs were heterogeneous in size, shape and composition, i.e. with regard to stromal percentages and amount of tumour epithelial cells, necrosis and/or mucin, both between as well as within individual patients. Figure 2 illustrates all of the measurements performed for each positive lymph node and their LNMs.

**Table 2:** Patient characteristics

Variable (unit)	Result (*n* = 45)
Age (years)	70 (55–78)
Gender (male, no.–perc.)	23 (51)
Total lymph nodes per patient (no.)	3 (2–5)
Total positive lymph nodes per patient (no.)	2 (1–3)
Histology (no.–perc.)	
-Adenocarcinoma -Mucinous adenocarcinoma	38 (85) 7 (15)

Baseline characteristics are presented in medians with interquartile range or numbers with frequencies

**Table 3:** Lymph node characteristics

Variable (unit)	Result (*n* = 126)
Diameter positive lymph node (mm)	6.4 (4.9–9.6)
Diameter LNM (mm) -Micrometastases (no.-perc.)	5.2 (2.8–8.2) 17 (13.5)
Area positive lymph node (mm^2^)	24.5 (12.8–52.6)
Area LNM (mm^2^)	13.3 (4.1–34.1)
Ratio area LNM: lymph node (%)	63.4 (31.5–79.0)
TSR-c (%)	35 (20–60)
TSR-t (%)	20 (10–30)
TSR-c category (no.–perc.)	
-Stroma-low -Stroma-high -Not applicable	56 (44.4) 35 (27.8) 35 (27.8)
Area adjusted TSR-c annotation (mm^2^)	1.0 (0.4–1.7)
Area of stroma in LNM (mm^2^)	2.1 (0.5–5.2)

*LNM* lymph node metastasis; *TSR-c* tumour-stroma ratio—conventional scoring method; *TSR-t* tumour-stroma ratio—total percentage of stroma; TSR categories: stroma-low (≤ 50% stroma) or stroma-high (> 50% stroma). Baseline characteristics are presented in medians with interquartile range or numbers with frequencies

### Tumour-stroma analysis

**Fig. 3:**
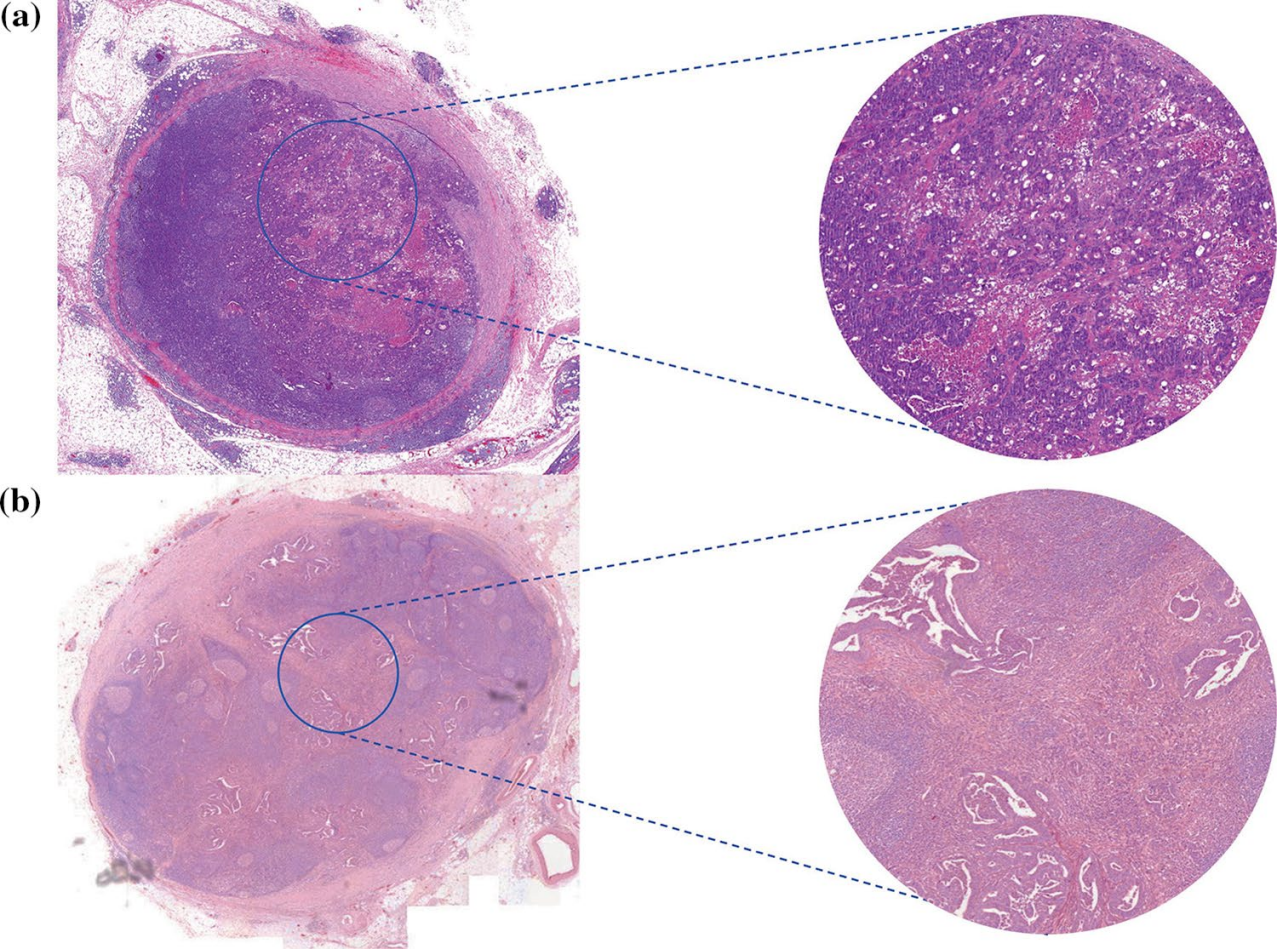
Positive lymph nodes containing a LNM with TSR-c annotations and corresponding 3.14mm^2^ highlighted areas: **a** Stroma-low metastasis. **b** Stroma-high metastasis

For the TSR-c score, the third, independent observer (PS) was decisive in 5 of the positive lymph nodes (4%). In the rest of the cases and the TSR-t, the scores were similar or agreement was reached, leading to an excellent overall Cohens’ kappa of > 0.9. The median TSR-c percentage of all the LNMs was 35% (IQR 20–60%). A total of 56 of the LNMs was defined as stroma-low (44%) and 35 (28%) were stroma-high, while in 35 cases (28%), the TSR-c method could not be applied according to the requirements of the guideline for scoring as described by van Pelt et al.[29], e.g. due to the limited size of tissue sample. In the latter group of LNMs, a median adjusted TSR-c annotation area of 1.0 mm^2^ (IQR 0.4–1.7 mm^2^) was used. Figure 3 shows a stroma-high and a stroma-low LNM and the corresponding TSR-c annotations. The median TSR-t score of all the lymph nodes was 20% (IQR 10–30%). A total of 3 (2%) of all analysed LNMs contained < 5% tumour stroma, the rest all were scored with a sufficient amount of stroma, i.e. TSR-t ≥ 5%.

### Subgroup analysis of positive lymph nodes using the radiology cut off

A total of 32 of the 126 positive lymph nodes (25%) had a diameter smaller than the 5.0 mm radiology cut-off value. In these nodes, the median TSR-c of the LNM was 30% (IQR 10–40%), and the median TSR-t was 20% (IQR 10–30%). Furthermore, a total of 52 of the 126 positive lymph nodes (41%) had an *area* of less than 19.6 mm^2^. A median TSR-c of 30% (IQR 12.50–60%) and TSR-t of 20% (IQR 10–30%) was observed in this subgroup. In all 32 small but positive lymph nodes with the criterion of a diameter of less than 5.0 mm, and in 51 positive lymph nodes covering an area of less than 19.6 mm^2^ (98%), a clear metastatic lesion of ≥ 5% total stroma was observed.

### Correlations between positive lymph nodes and the LNM

**Fig. 4:**
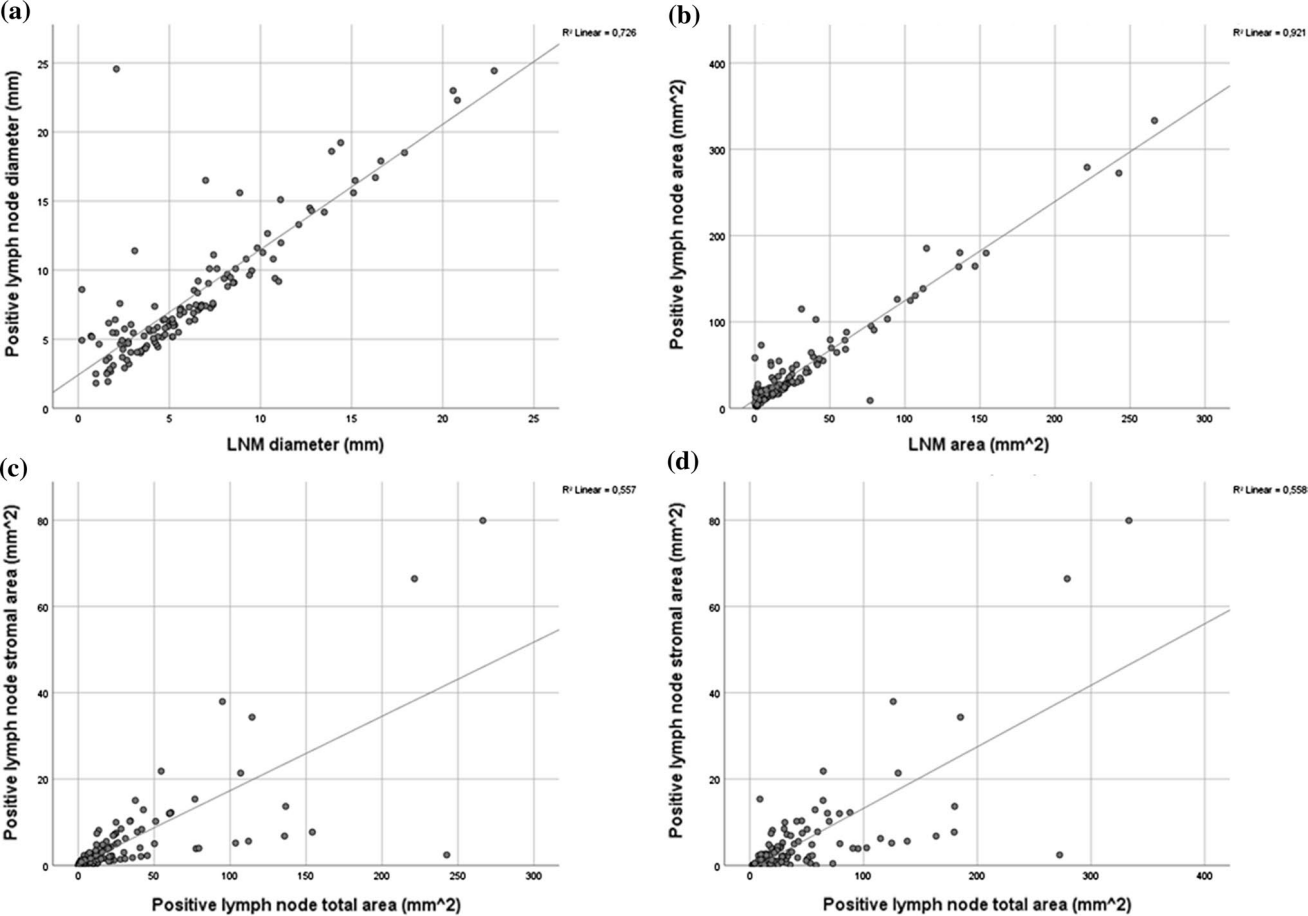
Overview of the different correlations, portrayed by scatterplot diagrams: **a** LNM diameter and positive lymph node diameter (*r* = 0.852, *p* < 0.001). **b** LNM area and positive lymph node area (*r* = 0.960, *p* < 0.001). **c** LNM stromal compartment area and LNM area (*r* = 0.746, *p* < 0.001). **d** LNM stromal compartment area and positive lymph node area (*r* = 0.747, *p* < 0.001)

Correlations between the positive lymph nodes and their respective LNMs were assessed, regarding size and stromal amount, to ascertain whether on current and future imaging, assumptions regarding lymph node size and potential malignancy could be made. Here, correlations were observed between the sizes of the positive lymph node and their LNM; diameters and areas of the lymph node and that of the LNM were significantly positively correlated (*r* = 0.852, *p* < 0.001 and *r* = 0.960, *p* < 0.001, respectively), i.e. that the larger the size of the positive lymph node was, a larger LNM was observed. The area of the amount of stroma in the LNM and the total area of the LNM showed a significant positive correlation as well (*r* = 0.746, *p* < 0.001). Moreover, the LNM stromal compartment area was correlated to the total positive lymph node area (*r* = 0.747, *p* < 0.001) and diameter (*r* = 0.612, *p* < 0.001). See Fig. 4 for an overview of the scatterplots showing these correlations.

## Discussion

This study provides an overview of histopathological features of positive lymph nodes, their respective LNMs and especially the tumour stroma within, in stage III CRC patients. First, in line with previous literature as well, we found that nearly all LNMs contained tumour stroma, despite morphological heterogeneity regarding size and proportional composition of non-stromal components [42–44]. Interestingly, all the positive lymph nodes smaller in diameter than 5.0 mm (32 out of 126, 25%), which could potentially have been missed with conventional imaging using the cut-off value as stated by guidelines, contained a sufficient amount of tumour stroma as well, i.e. a TSR-t score ≥ 5%.

Furthermore, we have shown that positive lymph node size positively correlates with the size of their respective LNMs, in diameter as well as in area, i.e. that larger positive lymph nodes contain larger LNMs. Both the lymph node and LNM area correlate with the absolute amount of stroma in the LNM, measured in mm^2^ as area. This means that, as positive lymph nodes are larger, a higher absolute amount of stroma can be expected. We have also assessed the proportional composition of LNM, i.e. the proportion of stroma versus other LNM components, e.g. tumour epithelial cells, mucin and/or necrosis, and have shown that the TSR does neither correlate with lymph node diameter nor with LNM diameter or area. In other words, both small and large positive lymph node or LNM may be proportionally stroma-high or stoma-low. These data may at first glance seem contradicting, but this means that, as LNM get larger, the absolute amount of stroma increases, independent of their proportional composition (stroma vs. other components), which is supported by other research [45].

It remains unknown whether the high proportion of stroma in positive lymph nodes will lead to improved detection with FAPI PET/CT, since literature lacks prospective studies with imaging and pathology correlation. Still, in molecular studies, FAP is strongly expressed in the CAFs, highly prevalent in stroma-high tumours, which could be an indication of a high number of binding spots for FAPI [13, 19]. Therefore, positive lymph nodes containing CAFs could potentially be visualized better, which correlates with previous findings, where the novel FAPI PET/CT scan has shown to improve detection of positive lymph nodes using this more targeted tracer in comparison to the standard FDG PET/CT, even leading to upstaging [21–25].

However, the CAF group makes up a heterogeneous cell population. FAP expression is mainly used as marker to localize and determine the CAFs, but there are many different subtypes of CAFs involved in the process of metastasizing, with various markers. Moreover, although minimally, FAP is expressed by more cells than the CAFs exclusively, e.g. the quiescent stromal cells in other tissue types, leading to some background uptake [46–48]. In addition, it has been demonstrated that inflammation or radiation- and surgery-induced fibrosis can show uptake of FAPI as well [18, 22, 24]. FAPI PET/CT scans may thus give false positive results, necessitating some precaution in evaluating previous study results. Moreover, although recent research distinguishes pathological substrate due to differences in uptake of FAPI, more prospective clinical studies for corroboration of imaging to histopathology need to be initiated as well [18, 22, 23, 40].

There are some limitations to this study. First, the radiological cut off of 5.0 mm in diameter used for analysis is not the cut off used in pathology. In pathology standards, lymph nodes containing only isolated tumour cells (< 0.2 mm) are seen as negative nodes, whereas lymph nodes with a LNM larger than 0.2 mm are classified as positive [8]. Moreover, this cut-off value used in radiology likely does not correspond exactly to the pathological 5.0 mm, since the short axis diameter on imaging is fully visible in a three-dimensional plane, whereas on a pathology diagnostic slide, the two-dimensional tissue is arbitrarily prepared. More prospective studies on correlations with radiological and pathological findings are, however, currently initiated for improved assessment. Second, in almost a third of cases, the TSR-c method could not be applied, e.g. due to limited size of the tissue sample or insufficient viable tissue in the LNM. The standard annotation size was adjusted until the rules were met as stated by the guideline by van Pelt et al. [29]. Moreover, although the TSR has also been proven to be of prognostic value in mucinous carcinomas, which have the potential to make as much stroma as adenocarcinomas without a mucinous component, it is not known how the mucin reacts to the FAPI tracer [28, 29]. Lastly, no additional immunohistochemistry was performed on the diagnostic slides, thus the actual presence of FAP on the CAFs was not verified in this work. Since there are many different CAF subtypes with various markers, this potentially could have led to an overestimated number of CAFs and thus FAPI uptake. For optimal corroboration of results, future studies should hence include immunohistochemistry with specific FAP staining.

Only patients with stage III colon cancer who underwent a curative resection without neoadjuvant therapy were included in this study. Therefore, the outcomes cannot be applied to patients receiving neoadjuvant chemotherapy or (chemo)radiotherapy, most commonly applied in rectal cancer patients [49, 50]. Neoadjuvant treatment alters the stromal component, potentially leading to fibrosis with aberrant, less detectable CAFs and thus a disrupted image of the tumour in resection material [51, 52]. In turn, this could cause discrepancies between imaging and histopathology results. Previously, it was found that the TSR is of significant value in predicting therapeutic response of neoadjuvant treatment in oesophageal and breast cancer, with a worse response in stroma-high tumours than stroma-low tumours [34, 35]. It would be interesting to evaluate these treated patients primary tumours and positive lymph nodes, including FAPI PET/CT imaging and biopsy beforehand and subsequent analyse the associated histological slides and the tumour stroma with additional immunohistochemistry, as current literature is lacking herein.

This is, to the best of our knowledge, the first article describing histopathological tumour-stromal features of LNMs in CRC patients in detail. In LNMs, stroma is abundantly present, serving as a potentially good substrate for FAPI PET/CT, even in small positive lymph nodes. Hence, the first step for a theoretical framework is taken for new standardization of imaging in cancer. The next step is to correlate tracer uptake in positive lymph nodes on imaging with FAPI PET/CT scanning to the histopathology results in prospective, clinical studies.

## Data Availability

The dataset analysed during this study is available from the corresponding author upon reasonable request.

## Abbreviations

CAF: Cancer-associated fibroblast
CRC: Colorectal cancer
CT: Computed tomography
FAP: Fibroblast activation protein
FAPI: Fibroblast activation protein inhibitor
FDG: Fluorodeoxyglucose
H&E: Haematoxylin and eosin
IQR: Interquartile range
LNM: Lymph node metastasis
MRI: Magnetic resonance imaging
PET: Positron emission tomography
TNM: Tumour-node-metastasis
TSR: Tumour-stroma ratio
TSR-c: Conventional tumour-stroma ratio
TSR-t: Total tumour-stroma ratio
